# An Immunohistochemical Study of β-catenin Expression and Immune Cell Population in Metastatic Carcinoma to the Liver

**DOI:** 10.3389/pore.2021.1609752

**Published:** 2021-06-04

**Authors:** Kwan-Yung Au, Regina Cheuk-Lam Lo

**Affiliations:** ^1^ Department of Pathology, The University of Hong Kong, Pok Fu Lam, Hong Kong; ^2^ State Key Laboratory of Liver Research, (The University of Hong Kong), Pok Fu Lam, Hong Kong

**Keywords:** liver, β-catenin, CD8, tregs, metastatic carcinoma

## Abstract

The liver is the commonest site of cancer metastasis. In this study, we asked whether the immune tumor microenvironment in liver metastases was governed by the β-catenin activation status of the tumor. To this end, we analyzed CD8 and FoxP3 immunohistochemical expression against β-catenin expression status of the tumor in a cohort of 52 liver samples with metastatic carcinoma. The results showed that colorectal primary constituted the largest proportion of metastatic carcinoma showing β-catenin overexpression. Intra-tumoral CD8 count was lower and FoxP3 count was higher when compared with the non-tumoral liver parenchyma. β-catenin overexpression was associated with a lower CD8 count in the tumor region (*p* = 0.003). In summary, our findings are in support of an altered immune tumor microenvironment vs. the non-tumor liver tissues in the metastatic site. Suppression of CD8 count was associated with activated Wnt/β-catenin signaling in the metastatic tumor.

## Introduction

The tumor microenvironment plays a crucial role in cancer cell survival and progression. Immune cells constitute a key component of the tumor microenvironment and it has been known that the immune system exhibits anti-tumor response [1]. Intrinsic differences in the immune microenvironment among different cancer types could be partly explained by genetic regulators that modulate the immune microenvironment. The most notable example is canonical Wnt pathway, of which activation was shown to correlate with immune exclusion across human cancer types and was identified as an attributing factor for “immune-cold” tumor microenvironment [2].

β-catenin signaling regulates immunosurveillance mainly through modulating immunogenicity of tumor cells or anti-tumor immune response [3]. For the latter, CD8^+^ T cell, regulatory T (Treg) cells and NK/T cells are the major effectors [3, 4]. The canonical Wnt pathway is commonly deregulated in many types of human cancers. One major mechanism contributing to pathway activation is mutation of intrinsic components of the pathway. The incidence of mutation differs among cancer types. For instance, mutations of key members in the Wnt/β-catenin pathway were identified in 90% of colorectal cancer; while the incidence was 18–44% in hepatocellular carcinoma, and rare in pancreatic ductal adenocarcinoma [5]. Apart from this, the signaling cascade can be activated via ligand binding, overexpression of intrinsic molecular targets in the pathway, or aberrant expression of extrinsic molecular regulators. In this regard, we previously identified Cripto-1 and SOX9 as activators of the Wnt/β-catenin signaling cascade in hepatocellular carcinoma [6, 7]. Albeit the various mechanisms, the common pivotal step is the stabilization of β-catenin protein in the cytoplasm followed by nuclear translocation which triggers transcription of a wide array of gene targets [8].

Cancer metastasis is a multi-step process involving local migration/invasion, intravasation, extravasation and colonization at metastatic site. Studies in recent years have shown that cancers facilitate their progression via modulating the milieu of the metastatic sites [9, 10]. The liver is the commonest site of metastasis. In this study, we hypothesized that the immune tumor microenvironment in liver metastases was governed by the β-catenin activation status of the tumor. We aimed at analyzing the immune cell population (CD8 and FoxP3, a marker for Treg cells) by means of immunohistochemistry (IHC); and identifying associations of the immune population patterns with the β-catenin status and origin of the tumor.

## Materials and Methods

### Clinical Samples

The HCC clinical samples came from the pathology archive in Queen Mary Hospital, a tertiary center in Hong Kong. Use of clinical samples was approved by Institutional Review Board (HKU/HA HKW IRB Ref. UW12–525). Liver resection specimens from year 2014–2020 were retrieved from the pathology database at Department of Pathology, Queen Mary Hospital. Fifty-six consecutive cases were initially identified. Histological sections were reviewed by a pathologist (R.C.L.) to confirm the diagnosis. Tumor and non-tumoral liver tissue sections were selected for each case. Four cases were excluded due to inadequate non-tumoral liver tissue or unavailable paraffin blocks.

### Immunohistochemical Staining and Analysis

IHC was performed on formalin fixed paraffin embedded (FFPE) sections using labeled horseradish peroxidase (HRP) method as we previously described [6, 7]. FFPE sections were deparaffinized in xylene and rehydrated in a series of graded ethanol to water. Heat antigen retrieval was performed with Tris-EDTA (pH 9.0) for 15 min using TissueWave™ two microwave processor (Thermo Scientific). Endogenous peroxidase activity was blocked using 3% H_2_O_2_ and serum-free protein block (Dako). Antibodies against human antigen β−catenin, CD8, and forkhead box P3 (FoxP3) were used. Sections were incubated at 4°C overnight with primary antibody CD8 (ab17147, 1:75, Abcam), FoxP3 (ab20034, 1:50, Abcam) or β−catenin (610153, 1:400, BD Biosciences) followed by incubation at room temperature with HRP−conjugated secondary antibody (Dako). Signals were then developed using the DAB + substrate chromogen system (Dako) and counterstained with hematoxylin. Immunohistochemical expression for β-catenin staining in tumor cells was assessed as previously described elsewhere [11]. Nuclear and/or cytoplasmic expression was taken as overexpression. For CD8 and FoxP3, expression (membranous for CD8 and nuclear for FoxP3) in lymphocytes was quantified as previously described [12, 13]. The number of positive-staining cells in five randomly selected high-power fields (Nikon Eclipse 50i 22 mm) were manually counted. The mean value was adopted for subsequent statistical analysis for each case (defined as “CD8 count” or “FoxP3 count”).

### Statistical Analysis

Clinicopathological parameters and IHC scores were analyzed using Mann-Whitney *U*-test. Correlation between CD8 and FoxP3 was analyzed using Pearson’s correlation. Statistical significance was analyzed using Prism 8 software (GraphPad).

## Results

### Clinicopathological Features of the Clinical Cohort

A total of 52 cases were included in the study comprising 31 male and 21 female patients ranging from 22 to 79 years of age. The primary sites of the tumor were detailed in Table 1. Among the 52 cases, 30 belonged to metastatic colorectal carcinoma; 22 cases were metastatic carcinoma from other sites including the breast, esophagus, nasopharynx, etc.

**TABLE 1:** β-catenin expression status according to primary site of tumor.

Primary site	No. of cases in cohort	No. of cases showing β-catenin overexpression (% in brackets)
Colon	30	27 (90%)
Breast	5	3 (60%)
Esophagus	3	3 (100%)
Nasopharynx	3	0 (0%)
Lungs	2	1 (50%)
Kidney	2	0 (0%)
Female genital tract	2	0 (0%)
Pancreatobiliary	2	1 (50%)
Salivary gland	1	1 (100%)
Adrenal gland	1	0 (0%)
Stomach	1	1 (100%)
Total	52	37

### β-catenin Expression Status of the Metastatic Tumors

**FIGURE 1:**
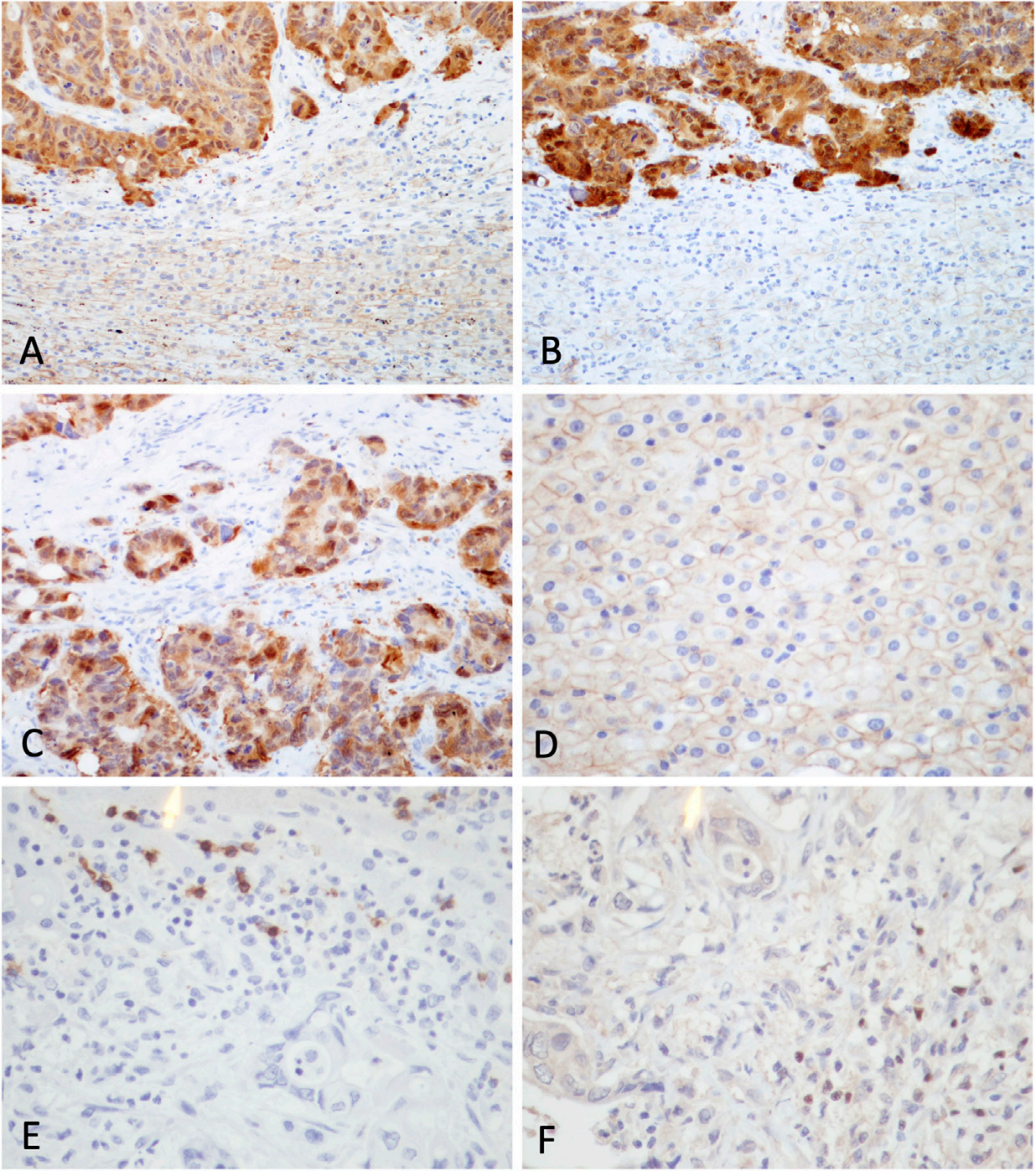
Immunohistochemical staining for β-catenin, CD8 and FoxP3 in clinical samples. **(A)** and **(B)** β-catenin staining at interface between tumor and non-tumor region from representative samples showing β-catenin overexpression (original magnifications: × 200). **(C)** Positive nuclear and cytoplasmic staining was observed in tumor cells. **(D)** Hepatocytes in non-tumor region demonstrated membranous staining. Expression of **(E)** CD8 and **(F)** FoxP3 in a case of metastatic carcinoma (original magnifications: × 400).

On immunohistochemical analysis for β-catenin staining, hepatocytes in the non-tumoral region demonstrated membranous staining. No staining was identified in the cytoplasm or the nucleus. Among the 52 cases, 37 were showed β-catenin overexpression in the tumor cells (Figures 1A–D). Among 37 cases, 27 were metastatic colorectal carcinoma. Other non-colorectal cases showing frequent β-catenin overexpression included metastatic carcinoma from the breast (3 of 5) and esophagus (3 of 3) (Table 1).

### Immune Cell Population in Tumor and Non-tumor Liver Tissue

**FIGURE 2:**
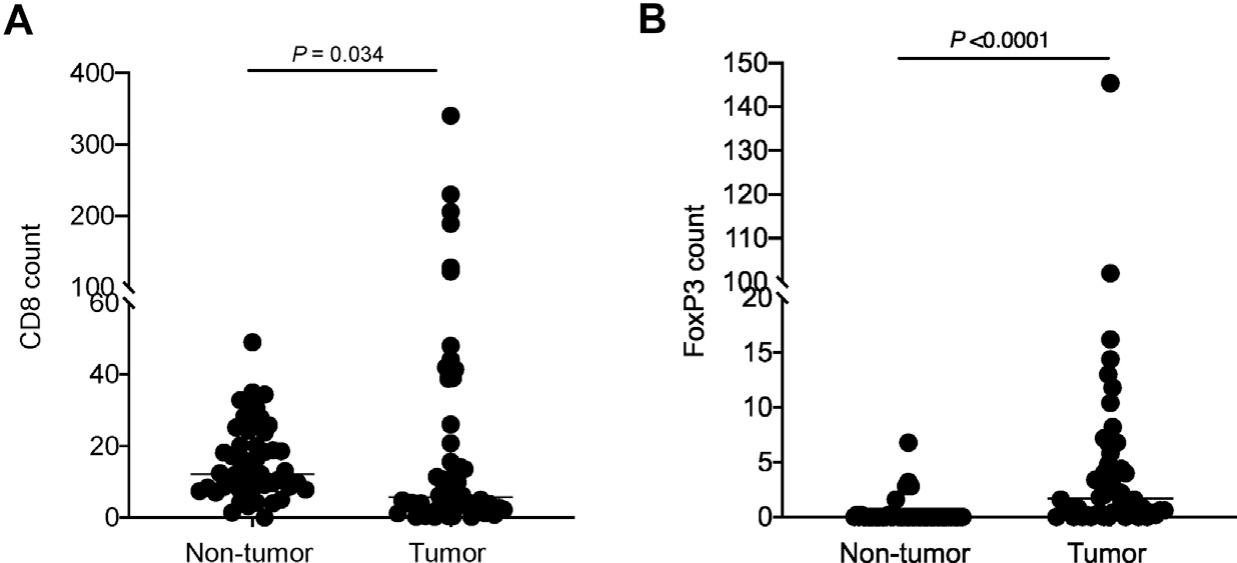
Comparison of **(A)** CD8 count and **(B)** FoxP3 count in tumor vs. non-tumor regions in the cohort. Each dot represents data point from one case; horizontal line represents the median.

The CD8 count by immunohistochemical staining ranged from 0.2–340 per HPF (median: 5.7 per HPF) in the tumor region and 0–49 per HPF (median: 12.2 per HPF) in the non-tumor region. The FoxP3 count ranged from 0–145.5 per HPF (median: 1.7 per HPF) in the tumor region and 0–6.8 per HPF (median: 0 per HPF) in the non-tumor region (Figures 1E,F, 2A,B). Upon statistical analysis, on comparing the CD8 and FoxP3 counts in the tumor vs. non-tumor regions in the cohort, we observed a lower CD8 count (*p* = 0.034) and a higher FoxP3 count (*p*=<0.0001) in the tumor region (Figures 2A,B). Correlation of CD8 counts or FoxP3 counts between the tumor vs. non-tumor region was not statistically significant (Supplementary Figure S1A,B).

### Association Between β-catenin Expression Status and Immune Microenvironment

**FIGURE 3:**
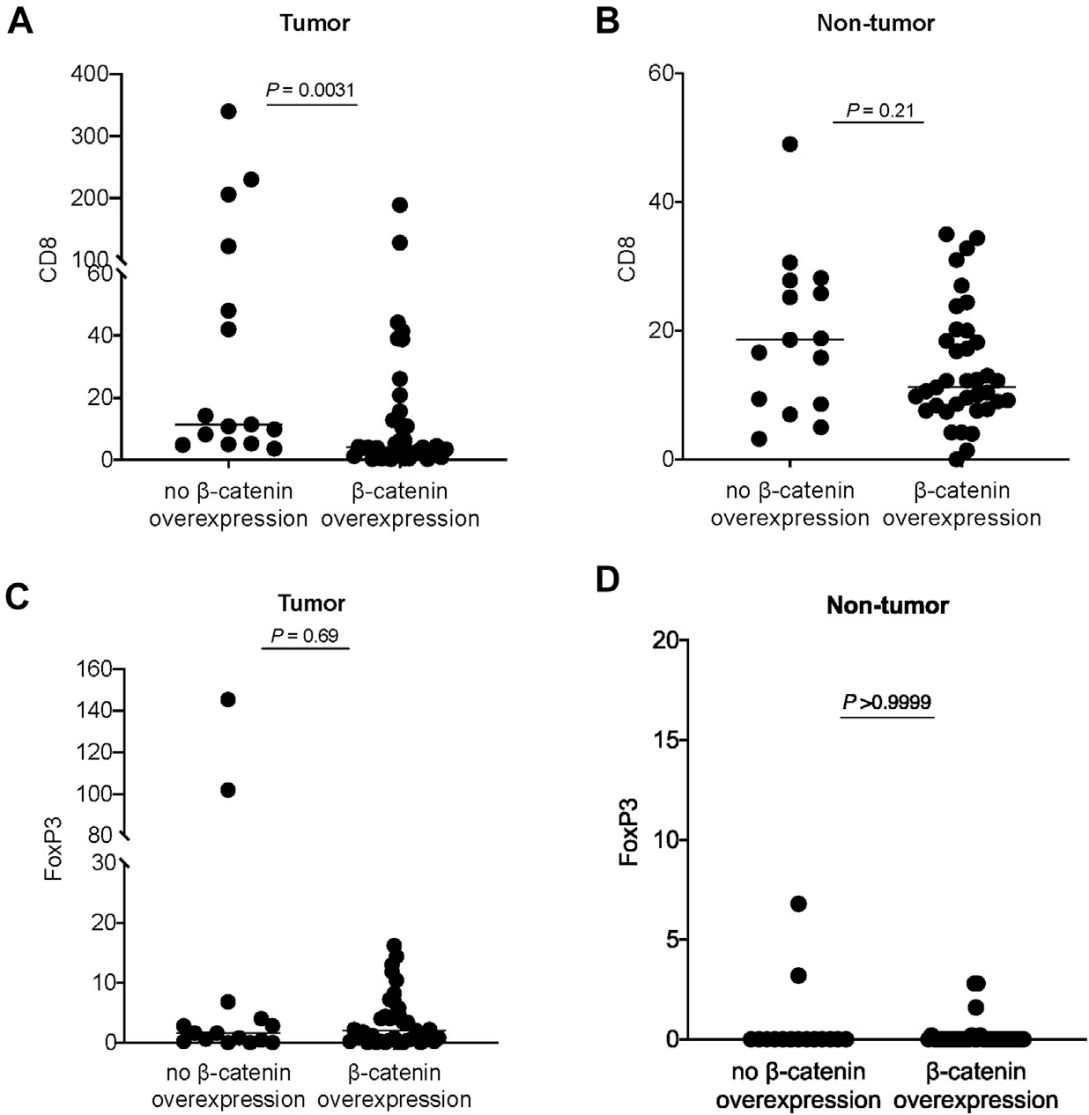
Analysis of β-catenin expression in tumor and CD8 count in **(A)** tumor region and **(B)** non-tumor region. Analysis of β-catenin expression in tumor and FoxP3 count in **(C)** tumor region and **(D)** non-tumor region. Each dot represents data point from one case; horizontal line represents the median.

β-catenin score was analyzed against the immune cell counts. In the tumor region, β-catenin overexpression was associated with a lower CD8 count (*p* = 0.003) (Figure 3A). A similar trend was observed in the non-tumor region but not reaching statistical significance (*p* = 0.21) (Figure 3B). No significant association was identified between β-catenin expression and FoxP3 count the tumor region or non-tumor region (Figures 3C,D).

## Discussion

We retrospectively analyzed the expression of β-catenin and immune cell markers CD8 and FoxP3 in a cohort of metastatic carcinoma in the liver. In our series, the colon constituted the top-ranking primary site, which was in line with findings from reported studies [14]. β-catenin overexpression was observed in 90% (27 of 30) samples from colorectal primary in this cohort, consistent with previous reports. Moreover, metastatic carcinoma from colorectal primary constituted 73% (27 of 37) cases showing β-catenin overexpression, suggesting that the colon is a major tumor origin accounting for β-catenin-overexpressing metastatic carcinoma to the liver.

On immune cell population, the CD8^+^ count was lower and FoxP3+ count was higher in tumor than non-tumor region, suggesting a deregulated immune microenvironment in the tumor region when compared with non-tumor region. Consistent with previous reports, β-catenin overexpression in tumor was associated with an immune-suppressive environment as defined by a lower CD8 count. β-catenin expression showed a trend of association with CD8 count in the non-tumor tissue (Figure 3B), suggesting that the effect of β-catenin pathway status in the tumor might extend to the non-tumor liver tissue in a certain degree.

Our study findings possibly provide some insights in management of cancers. Immune checkpoint inhibitors are implicated in the treatment of many advanced stage cancers. Concomitant targeting of β-catenin in some cancer types could potentially augment therapeutic effect. The relatively smaller cohort size is a limitation of our study. Besides, use of image analysis software for IHC staining could help to overcome the concern of reproducibility with the current single-observer approach. For future continuation work, previous studies demonstrated an association between in CD8 count and survival [15–17] and this correlation could be evaluated in an expanded cohort. In addition, extension of these analyses in metastatic cancers of non-epithelial origin could be considered.

## Data Availability

The original contributions presented in the study are included in the article/Supplementary Material, further inquiries can be directed to the corresponding author.
