# Supplementary figures and images for: An Immunohistochemical Study of β-catenin Expression and Immune Cell Population in Metastatic Carcinoma to the Liver
Source: Pathol Oncol Res. 2021 Jun 4;27:1609752. doi: 10.3389/pore.2021.1609752 (PMC8262218; doi:10.3389/pore.2021.1609752)

Supplementary Figure S1

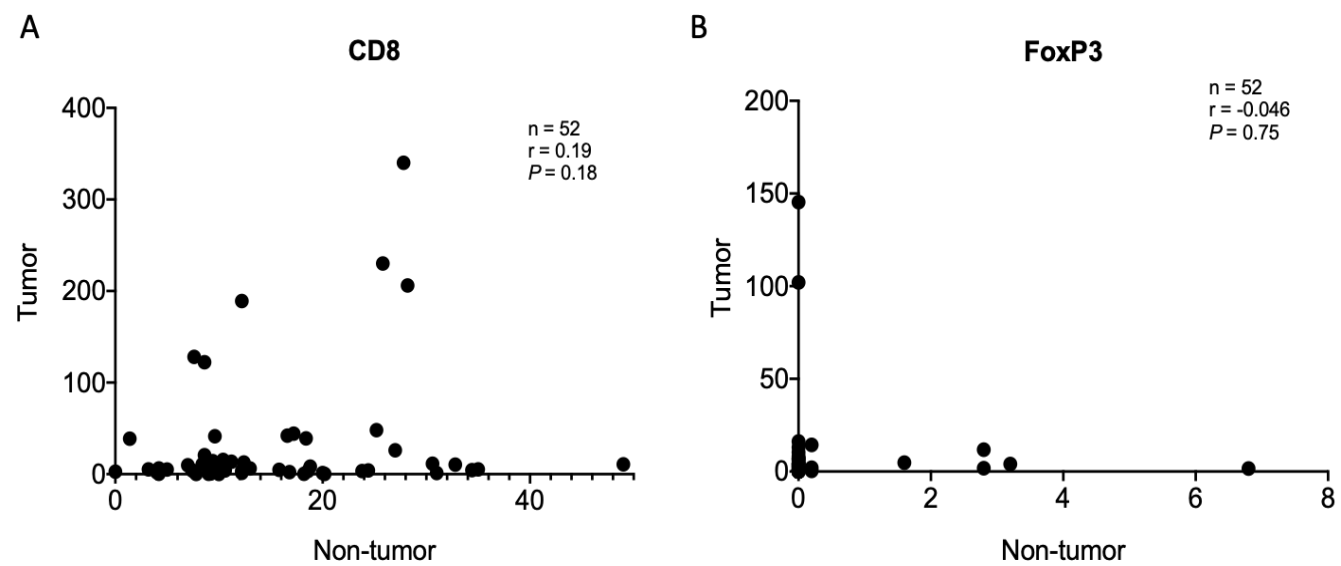

Supplement: Supplementary file 1 [file DataSheet1.PDF]
